# Reciprocating the Future: Exploring Natural Human Behaviors in Humanoid Social Robot Interaction

**DOI:** 10.1002/alz.094216

**Published:** 2025-01-09

**Authors:** Reilly Moberg, Arshia A Khan

**Affiliations:** ^1^ University of Minnesota Duluth, Duluth, MN USA

## Abstract

**Background:**

When designing cutting‐edge technology, particularly humanoid social robots, an essential consideration is understanding how individuals naturally engage in social interactions, shaping their relationships with technology and media.

**Method:**

In pursuit of insights into the application of natural human behavior, specifically reciprocation, in human‐robot interaction, an experiment involving 72 participants, involving facial electromyography, focusing on zygomatic and corrugator muscles, served as a tool to gauge users' emotional valence during interactions. The study assessed users' willingness to reciprocate a favor and measured compliance by tracking the number of raffle tickets purchased by users at the robot’s request. Events were recorded during the participant’s interaction with Pepper the humanoid robot, analyzing facial EMG data in five‐second intervals to capture changes in muscle activity before and after key events. The study comprised three scripted events, including the participant’s first sighting of Pepper, Pepper offering water (favor condition only), and a simulated glitch on Pepper’s tablet. Post‐interaction, participants filled out a final survey, contributing to the comprehensive examination of their experiences. The research involved 72 undergraduate students from diverse majors. The study aimed to assess initial impressions of humanoid robots, utilizing a phasic study design with a five‐second response time, aligning with previous research practices. The participants, largely lacking prior experience with social, service, or humanoid robots, provided valuable insights into human‐robot interaction dynamics.

**Results:**

In testing two hypotheses on participants' interaction with a Humanoid Social Robot (HSR), we found no significant differences in ticket purchases between favor and non‐favor conditions using statistical tests, including chi‐square and Mann‐Whitney U. However, an independent samples t‐test, after outlier removal, revealed a significant difference, supporting both hypotheses. Pearson’s correlation coefficients showed positive correlations between emotional responses, but no significant relation with self‐reported affect or ticket purchases. These findings enhance understanding of human‐robot interaction dynamics and inform future research directions.

**Conclusion:**

This study emphasizes the importance of incorporating natural human behaviors, like reciprocation, in designing humanoid social robots, indicating that initiating positive interactions significantly impacts users' compliance in subsequent interactions, advancing the field of human‐robot interaction with theoretical implications and proposing future research avenues despite acknowledged study limitations.

